# How Do Tourists’ Value Perceptions of Food Experiences Influence Their Perceived Destination Image and Revisit Intention? A Moderated Mediation Model

**DOI:** 10.3390/foods13030412

**Published:** 2024-01-26

**Authors:** Yijin Zhu, Liqun Zhu, Lisheng Weng

**Affiliations:** 1 College of Humanities & Social Development, Nanjing Agricultural University, Nanjing 210095, China; 2021110031@stu.njau.edu.cn (Y.Z.); zhulq@njau.edu.cn (L.Z.); 2 School of Tourism Management, Hubei University, Wuhan 430062, China; 3 Hubei Digital Culture and Tourism Research Institute, Hubei University, Wuhan 430062, China; 4 China Resources & Environment and Development Academy, Nanjing Agricultural University, Nanjing 210095, China

**Keywords:** food experience, perceived destination image, revisit intention, food tourism, Nanjing Impressions

## Abstract

The food experience is an important part of the tourism experience. Although it is crucial to comprehend the significance of tourists’ perception of food experiences, there is a scarcity of research investigating the impact of tasting local food on tourists’ perceptions and behaviors. This study employs structural equation modeling to empirically examine the relationship between tourists’ value perceptions of food experiences, their perceived destination image, and their revisit intention. In addition, the moderating effects of tourists’ genders on the aforementioned relationships are also explored. A renowned restaurant brand, Nanjing Impressions, which specializes in offering the unique cuisine of the city of Nanjing, China, is chosen as the research case. A grand total of 500 questionnaires were distributed, and, out of these, 458 questionnaires were deemed legitimate and kept for further analysis. The results indicate that the functional, social, emotional, cultural, and health values of local food experienced by tourists have significant positive impacts on their perceived destination image and revisit intention. Moreover, perceived destination image partially mediates the relationships between tourists’ value perceptions of food experiences and their revisit intention. Gender is found to partially moderate the relationships between the proposed constructs. The current study offers noteworthy theoretical contributions and provides valuable practical suggestions for tourism destination managers.

## 1. Introduction

Food tourism is a crucial component of the overall tourism experience and significantly impacts the sustainability of the destination. When tourists are contented with a favorable and gratifying food experience, they establish profound and enduring recollections of the pleasant event. Pleasant memories contribute to the enhancement of tourists’ favorable attitudes towards the local food of a destination, and lead to positive travel outcomes, such as higher satisfaction levels or the intention to revisit the destination [1]. In addition, the food experience is not only an important factor influencing travel motivation and travel decisions, but also the attractiveness of a destination. Hence, it is crucial for destination marketing organizations and managers to thoroughly investigate tourists’ local food experiences to enhance their competitiveness and foster loyalty.

In recent years, there has been a consistent rise in food-related research within the domain of tourism and hospitality [2]. An area of interest within this topic pertains to the examination of tourists’ value perceptions of food experiences and their behavior [3]. Local food reflects the cultural and individual characteristics of a country, region, or person, and has the potential to improve the image of a destination [4,5]. The issue of how tourists’ value perceptions of food experiences affect their perceived destination image and behavior is extremely important. However, few studies have attempted to examine the interrelationships between tourists’ value perceptions of food experiences, perceived destination image, and their revisit intention.

Additionally, the effects of tourists’ value perceptions of food experiences on the aforementioned relationships should be investigated based on tourists’ genders, as there is a significant disparity in food experiences between male and female tourists [6]. For example, in a study conducted by Dedeoglu et al. [7], it was discovered that men are predominantly swayed by their opinion of the price’s value when contemplating a return visit to a place, whereas women are primarily influenced by their perception of the quality. Nevertheless, the moderating effects of gender on the aforementioned relationships still remain unexplored [8].

The present study aims to fill the current research gaps. Specifically, the research objectives of this study are (1) to examine the impacts of tourists’ value perceptions of food experiences on their perceived destination image and revisit intention, and (2) to explore the moderating effects of gender on the relationships between tourists’ value perceptions of food experiences, perceived destination image, and their revisit intention. Nanjing Impressions is chosen as the research case due to its renowned reputation for providing genuine traditional cuisine and its representation of unique Nanjing folk customs.

## 2. Literature Review and Development of Hypotheses

### 2.1. Tourists’ Value Perception of Food Experiences

The concept of perceived value has been extensively examined within the domain of tourism research [9]. Zeithaml [10] defines perceived value as the consumer’s overall assessment of a product’s utility based on a trade-off between benefits and costs. Murphy et al. argued that the concept of perceived value can be seen as the amalgamation of the perceived quality and a matching price associated with a certain destination [11]. Hence, the perception of value among tourists refers to the comprehensive assessment of the degree to which items or services fulfill their tourism requirements in specific situations, through a comparative analysis of their advantages and disadvantages [12]. Recently, the act of sampling indigenous gastronomy has gained significance among travelers, leading to the emergence of food tourism as a highly appealing, vibrant, and innovative facet of the tourism industry. According to Amin and Roy [13], travelers are able to fulfill various demands, such as material, cultural, social, and status demands, through the experience of consuming delectable cuisine throughout their trips. The perception of food by tourists plays a crucial role in shaping their overall perception of a destination [14]. It significantly affects tourists’ food experiences and revisit intentions towards the destination [15].

The dimensions of the perceived value of food experiences have been divided in a number of different ways [15,16,17,18]. One of the more frequently employed categorizations stems from the research of Hsu et al. [19]. These researchers measured the perceived value of the food experience using seven distinct dimensions, namely, functional value, social value, emotional value, epistemic value, cultural value, health value, and conditional value. The functional value of a product pertains to the usefulness that consumers experience from its attributes and functional features, which significantly influences consumers’ purchasing decisions [20]. Social value encompasses four levels of relational usefulness: the enhancement of the consumer’s personal cognitive capacities, and the formation of social connections, the acquisition of social acknowledgement, and the ultimate attainment of self-fulfillment [21,22]. Emotional value refers to the range of positive emotions, such as enthusiasm, relaxation, and pleasure, that visitors experience when they engage with the aesthetics and enjoyability of food. This leads to the emotional satisfaction of visitors [23,24]. Moreover, the study conducted by Sweeney and Soutar in 2001 discovered that epistemic value significantly impacts tourists’ food experiences at the destination [15]. Sampling regional cuisine offers a unique opportunity for travelers to appreciate its perceived value by fulfilling their curiosity and need for knowledge [25,26]. Cultural value is defined as the utility of a food experience that satisfies the desire of tourists to learn about the traditions and customs of a destination and to gain new knowledge and cultural cognition [27]. Health value refers to the perceived utility of improved physical and mental health acquired by the consumption of food which provides balanced nutrition and good health to the tourists [28]. According to Kim and Eves [23], using local ingredients in food not only alleviates hunger, but also ensures freshness and promotes better health. Conditional value is a measure of the perceived utility according to a specific situation or surrounding environment [25].

### 2.2. Relationship between Tourists’ Value Perception of Food Experiences, Perceived Destination Image, and Revisit Intention

A perceived destination image refers to the collective beliefs, impressions, and subjective perceptions that travelers have about a certain place [29,30]. The significance of imagery is crucial in the interaction between tourists and their destination, and it is a fundamental aspect that influences tourists’ satisfaction and post-trip behavior [9,31,32]. The perception of a destination’s image is significantly influenced by local food, which is considered an essential factor that tourists must experience. The act of consuming local food is believed to have a favorable influence on the perceived image of a destination [33]. For example, Ding and Jiang [34] examined the impact of perceived restaurant innovativeness on Generation Z tourists. They found that the tourists’ perception of menu innovativeness, technology-based service innovativeness, and experiential innovativeness were the key factors influencing their perception of the image of the destination. In addition, Lu and Hu [35] discovered that there is a significant influence of visitors’ perceived value on the destination’s image when examining the connection between the tourism experience, perceived value, and destination image of Macau tourists. Based on the findings of previous research, the following hypotheses are proposed:

**H1.**  *Tourists’ value perceptions of food experiences have a significant positive influence on perceived destination image.*

**H1a.**  *Tourists’ perceptions of the functional value of food experiences have a significant positive effect on perceived destination image.*

**H1b.**  *Tourists’ perceptions of the social value of food experiences have a significant positive effect on perceived destination image.*

**H1c.**  *Tourists’ perceptions of the emotional value of food experiences have a significant positive effect on perceived destination image.*

**H1d.**  *Tourists’ perceptions of the epistemic value of food experiences have a significant positive effect on perceived destination image.*

**H1e.**  *Tourists’ perceptions of the cultural value of food experiences have a significant positive effect on perceived destination image.*

**H1f.**  *Tourists’ perceptions of the health value of food experiences have a significant positive effect on perceived destination image.*

**H1g.**  *Tourists’ perceptions of the conditional value of food experiences have a significant positive effect on perceived destination image.*

Revisit intention refers to the probability that a tourist will choose to return to a destination within a specific timeframe following their initial visit [36]. Researchers believe that the intention to revisit can be used in conjunction with specific behavioral situations to accurately predict the actual behavior of tourists [37]. Extensive research has been conducted to investigate the various factors that influence the intention of visitors to revisit. For example, Petrick [38] argued that the perceived value model can be used to explain the behavioral intentions of cruise passengers. Moreover, it was discovered by Fu et al. [39] that the behavioral intentions of travelers were positively influenced by the social, emotional, and spiritual aspects of perceived value. Similarly, previous research on food festivals and food consumption has demonstrated that positive culinary perceptions and experiences are significant predictors of tourists’ willingness to revisit [40,41]. Research on food tourism in Africa has revealed that the food consumption experience and behavioral response of international travelers are influenced by their perceptions of the attributes and value of local food [42]. Tourists whose perception of a food experience’s value is greater demonstrate a greater propensity to return [43,44]. As a result, a degree of agreement has been reached regarding the feasibility of assessing the efficacy of transmitting a visitor’s perceived value into their intention to return. The following hypotheses are thus proposed:

**H2.**  *Tourists’ value perceptions of food experiences have a significant positive influence on their revisit intention.*

**H2a.**  *Tourists’ perceptions of the functional value of food experiences have a significant positive effect on their revisit intention.*

**H2b.**  *Tourists’ perceptions of the social value of food experiences have a significant positive effect on their revisit intention.*

**H2c.**  *Tourists’ perceptions of the emotional value of food experience have a significant positive effect on their revisit intention.*

**H2d.**  *Tourists’ perceptions of the epistemic value of food experiences have a significant positive effect on their revisit intention.*

**H2e.**  *Tourists’ perceptions of the cultural value of food experiences have a significant positive effect on their revisit intention.*

**H2f.**  *Tourists’ perceptions of the health value of food experiences have a significant positive effect on their revisit intention.*

**H2g.**  *Tourists’ perceptions of the conditional value of food experiences have a significant positive effect on their revisit intention.*

There is substantial evidence to suggest that the image of a tourist destination is highly correlated to a tourist’s intention to revisit. Throughout the decision-making and post-decision behavior of tourists, including their travel experience, satisfaction, and future behavioral intentions, the impact of destination image is pervasive [23,30,45]. A positive perception of the destination’s image by visitors serves as the primary impetus for their intention to revisit, according to the findings in the literature [46,47]. Similarly, a study conducted by Zhang et al. [48] found that country image and destination image influence the revisit intention through the mediating role of memorable tourism experiences. In addition, the study conducted by Khanh et al. [49] in Ba Ria-Vung Tau province, Vietnam, identified eight key factors that contribute to the destination image and influence domestic tourists’ intention to revisit. These factors include local food, infrastructure, variety seeking, accessibility, and entertainment. Therefore, the preceding findings lead to the formulation of the following hypothesis:

**H3.**  *A tourist’s perceived destination image has a significant positive influence on their revisit intention.*

The aforementioned study suggests that that tourists’ value perception of food experiences can impact their perceived destination image, and that the perceived destination image can influence tourists’ willingness to revisit. Thus, the perceived destination image may act as a mediating factor. Prior studies have extensively examined the mediating effect of the perceived destination image. For instance, Kim et al. [50] investigated the impact of the Korean TV series “Daeganggeum” on the perception of Korean food among inhabitants of Hong Kong, Taiwan, and Thailand. They found that this portrayal of Korean food had a significant influence on the overall image of the destination and also had a positive effect on the likelihood of tourists revisiting. A study on local food consumption by Hong Kong tourists yielded a similar finding, indicating that the tourists’ perception of food value in terms of quality, emotional appeal, and knowledge directly impacts their attitudes and perception of the destination image. This, in turn, significantly affects their likelihood of recommending the destination and their intentions to revisit [51]. Hence, it can be inferred that the perceived value of food experiences by tourists influences their willingness to revisit through the destination image. Based on the aforementioned findings, the following hypotheses are formulated:

**H4.**  *Perceived destination image mediates the influence of tourists’ value perceptions of food experiences on their revisit intention.*

**H4a.**  *Perceived destination image mediates the influence of tourists’ perceptions of the functional value of food experiences on their revisit intention.*

**H4b.**  *Perceived destination image mediates the influence of tourists’ perceptions of the social value of food experiences on their revisit intention.*

**H4c.**  *Perceived destination image mediates the influence of tourists’ perceptions of the emotional value of food experiences on their revisit intention.*

**H4d.**  *Perceived destination image mediates the influence of tourists’ perceptions of the epistemic value of food experiences on their revisit intention.*

**H4e.**  *Perceived destination image mediates the influence of tourists’ perceptions of the cultural value of food experiences on their revisit intention.*

**H4f.**  *Perceived destination image mediates the influence of tourists’ perceptions of the health value of food experiences on their revisit intention.*

**H4g.**  *Perceived destination image mediates the influence of tourists’ perceptions of the conditional value of food experiences on their revisit intention.*

### 2.3. The Moderating Role of Gender

Gender is a demographic factor that can influence how tourists perceive their environment and shape their perception of destination image [6,8,52]. Research has indicated that the impact of perceived value on tourists’ behavioral intentions can vary depending on gender. For instance, in a study on Iranian culinary tourism, researchers found that women have a greater level of experience compared to men in terms of enjoyment, freedom, power, and engagement with food [53]. In the same vein, Dedeoglu et al. [8] found that men are most influenced by their perception of the value of the price when considering revisiting a place, whereas women are primarily influenced by their perception of the quality of the place. By including gender as a moderating variable in our model, we researchers are able to expand upon the current theoretical framework about the perceived value of food experiences, perceived destination image, and tourists’ intentions to revisit. Based on the aforementioned empirical observations, the subsequent research hypotheses are offered:

**H5.**  *Gender moderates the influence of tourists’ value perceptions of food experiences on their perceived destination image.*

**H5a.**  *Gender moderates the influence of tourists’ perceptions of the functional value of food experiences on their perceived destination image.*

**H5b.**  *Gender moderates the influence of tourists’ perceptions of the social value of food experiences on their perceived destination image.*

**H5c.**  *Gender moderates the influence of tourists’ perceptions of the emotional value of food experiences on their perceived destination image.*

**H5d.**  *Gender moderates the influence of tourists’ perceptions of the epistemic value of food experiences on their perceived destination image.*

**H5e.**  *Gender moderates the influence of tourists’ perceptions of the cultural value of food experiences on their perceived destination image.*

**H5f.**  *Gender moderates the influence of tourists’ perceptions of the health value of food experiences on their perceived destination image.*

**H5g.**  *Gender moderates the influence of tourists’ perceptions of the conditional value of food experiences on their perceived destination image.*

**H6.**  *Gender moderates the influence of tourists’ value perceptions of food experiences on their revisit intention.*

**H6a.**  *Gender moderates the influence of tourists’ perception of functional value of food experience on their revisit intention.*

**H6b.**  *Gender moderates the influence of tourists’ perceptions of the social value of food experiences on their revisit intention.*

**H6c.**  *Gender moderates the influence of tourists’ perceptions of the emotional value of food experiences on their revisit intention.*

**H6d.**  *Gender moderates the influence of tourists’ perceptions of the epistemic value of food experiences on their revisit intention.*

**H6e.**  *Gender moderates the influence of tourists’ perceptions of the cultural value of food experiences on their revisit intention.*

**H6f.**  *Gender moderates the influence of tourists’ perceptions of the health value of food experiences on their revisit intention.*

**H6g.**  *Gender moderates the influence of tourists’ perceptions of the conditional value of food experiences on their revisit intention.*

**Figure 1:**
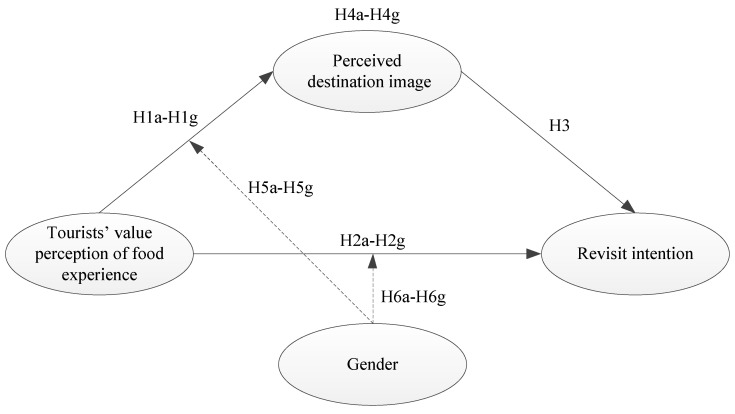
The conceptual framework of this study.

This study’s conceptual framework is illustrated in Figure 1.

## 3. Materials and Methods

### 3.1. Description of the Research Case

**Figure 2:**
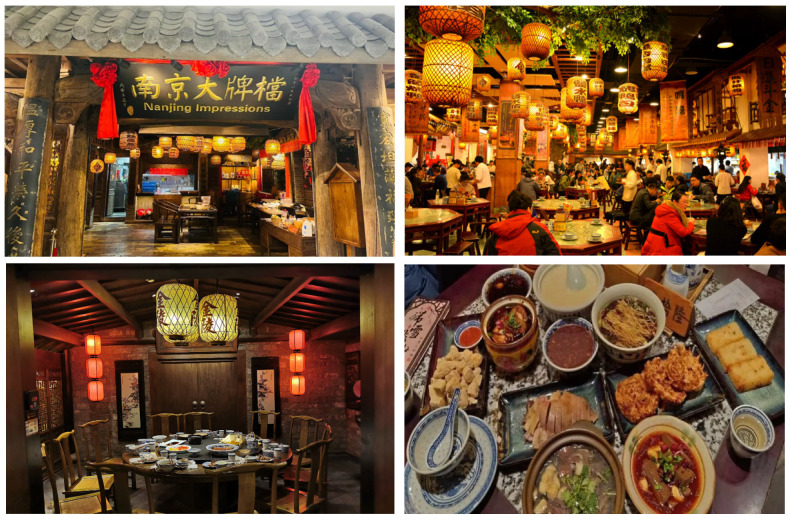
Photos of a Nanjing Impressions restaurant.

The selection of Nanjing Impressions as the research case is based on its reputation for offering authentic traditional cuisine and its embodiment of distinctive Nanjing folk customs (see Figure 2). Established in the year 1994, Nanjing Impressions is a renowned restaurant brand that specializes in offering the unique cuisine of the city of Nanjing. Its signature dishes include Duck Blood Vermicelli Soup, Jinling Roast Duck, etc. Nanjing Impressions has consistently received accolades such as being listed on the “must-eat restaurants list”, “popular food list”, and “favorable food list” of dianping.com. Dianping.com is a renowned and independent third-party consumer review site, boasting a user base of over 200 million active individuals in China. With a commendable track record, Nanjing Impressions has successfully established a network of 21 branches in Nanjing, thus solidifying its status as a highly sought-after specialty dining establishment among the majority of tourists visiting the city.

### 3.2. Questionnaire Design

The questionnaire utilized in this study is composed of three distinct sections. The initial section comprises two screening questions (Is it your first time visiting Nanjing? Is it your first time dining at Nanjing Impressions?), while the subsequent section pertains to the measurement of three fundamental variables, namely, the value perception of the food experience, the perceived destination image, and revisit intention. The third component pertains to the socio-demographic characteristics of the participants, encompassing variables such as age, gender, educational background, occupation, and income level.

The measurement methods of the three variables are all developed from previous scholarly investigations. The measurement of the value perception of the food experience was derived from the scholarly works of Hsu et al. [19]. The construct comprises seven distinct dimensions, encompassing a cumulative total of twenty-seven individual components. The seven dimensions are functional value (4 items, e.g., “There is a variety of menu choice”), social value (3 items, e.g., “Having the food in Nanjing Impressions obtain social approval”), emotional value (4 items, e.g., “The foods or the restaurants can make me feel happy”), epistemic value (3 items, e.g., “I want to seek out more information about foods in Nanjing”), cultural value (7 items, e.g., “It enables me to learn what this local food tastes like”), health value (4 items, e.g., “The foods or restaurants are hygienic”), and conditional value (2 items, e.g., “I would try the Nanjing duck blood vermicelli soup”). Perceived destination image was measured through an eight-item scale adapted from the research of Lertputtarak et al. [54]. A sample statement from this method is “Nanjing is a city full of opportunity for adventure”. The three-item scale utilized for the evaluation of respondents’ willingness to revisit was developed from the studies of Choe and Kim [18] and Bigné et al. [30]. An example question from this method is “I will come back to Nanjing in near future”.

The measurement of all responses was conducted using a five-point Likert scale, with response options ranging from “1 = Completely Disagree” to “5 = Completely Agree”. In order to facilitate comprehension among Chinese tourists, the original English questionnaire was initially translated into Chinese [55]. The phrasing of all the items was adjusted to align with the context of a Nanjing culinary encounter. Furthermore, a reverse translation was employed to ensure the precision of the translation [56]. A pilot study was carried out in June 2022, involving a sample of 8 tourists who had patronized Nanjing Impressions. Following the completion of the questionnaire, participants were asked to identify issues that posed challenges in terms of their comprehension of the questions or that generated controversy. The questionnaire was modified in response to the feedback received in order to enhance its comprehensibility.

### 3.3. Data Collection and Analysis

The research employed in-person surveys as a means of data collection over the months of July and August in the year 2022. Four research assistants who possess specific training in quantitative data collecting were recruited. Two screening criteria were utilized to ascertain the eligibility of respondents, with the aim of mitigating the impact of prior travel experiences [57]. The aforementioned standards are (1) that it is imperative for the individual to be on their inaugural visit to Nanjing, and (2) they must be partaking in a first-time dining experience at Nanjing Impressions. After verifying that the diners at Nanjing Impressions had completed their meals, they were asked to respond candidly to the questionnaire regarding their thoughts and experience. The participants were aided by research assistants who were there to address any inquiries during the survey, thus ensuring the integrity of the data collected. In this study, a total of 500 questionnaires were distributed and subsequently collected. The final analysis removed 42 questionnaires due to missing answers, resulting in a sample size of 458 that were eligible for analysis.

**Figure 3:**
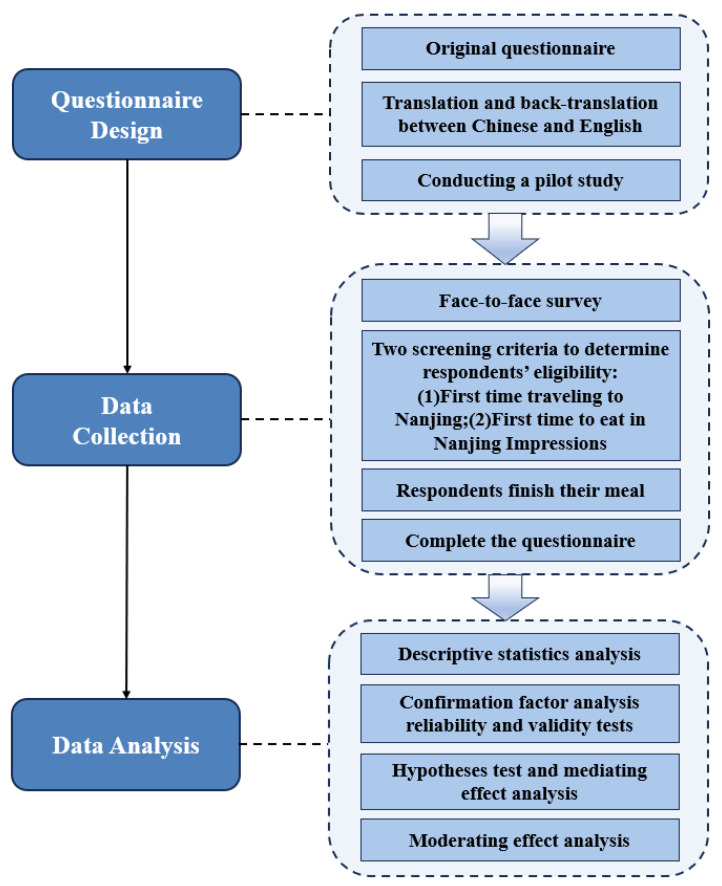
The technical roadmap for questionnaire design, data collection, and data analysis.

The data analysis in this study was conducted using SPSS 20.0 and Amos 21.0. The data analysis consists of four distinct stages. Initially, a descriptive statistics analysis was performed to investigate the demographic characteristics of the respondents. Next, the application of a confirmatory factor analysis (CFA) and reliability and validity testing was employed to assess the validity of the dimensions inside the model. Subsequently, hypotheses testing and mediation analysis were conducted to evaluate the hypotheses proposed in the previous section. Finally, a moderating effect analysis was performed to examine the moderating role of gender. The technical roadmap for the questionnaire design, data collection, and data analysis is illustrated in Figure 3 and outlined below.

## 4. Results

### 4.1. Sample Profile

The demographic characteristics of the sample are shown in Table 1. Among the participants, it was observed that 50.7% identified as male, while 49.3% identified as female, resulting in a balanced gender distribution. Approximately half (48.6%) of the participants fell between the age range of 18 to 35 years. A majority of the participants (54.6%) possessed a bachelor’s degree or a higher level of education. Furthermore, with regard to employment, the largest proportion of participants were engaged in business-related occupations, accounting for 43.9% of the total. This was followed by students and self-employed individuals, constituting 16.6% and 13.1% of the sample, respectively. Additionally, the income category ranging from CNY 6000 to CNY 7500 per month represented the largest number, accounting for 36% of the total population surveyed.

### 4.2. Measurement Model Testing

#### 4.2.1. Reliability Test and CFA

In order to ascertain the scientific integrity of the sample data, it is important to conduct a preliminary assessment to determine whether the sample data adhere to a normal distribution. The present analysis involved an examination of the skewness and kurtosis of the sample data utilizing SPSS 25.0. The findings indicated that the skewness coefficient exhibited a range of −0.982 to −0.231, while the kurtosis coefficient ranged from −0.898 to 0.736. These results suggest that the data were normally distributed [58,59,60].

The internal consistency of the scale was assessed using Cronbach’s alpha coefficient. The results are presented in Table 2. The scale demonstrated a high level of internal consistency, as indicated by its total Cronbach’s alpha coefficient of 0.941 [61,62]. Additionally, the individual variables within the scale exhibited Cronbach’s alpha coefficients ranging from 0.831 to 0.925. These findings suggest that the scale met the necessary criteria for hypothesis testing [63].

In addition, this study employed confirmatory factor analysis (CFA) to assess the measurement model [64]. Table 2 displays the model fit indices, which demonstrate that they meet the predefined threshold values (χ^2^/df = 1.141, NFI = 0.935, CFI = 0.991, TLI = 0.990, GFI = 0.926, AGFI = 0.913, RMSEA = 0.018, SRMR = 0.027) [65]. The findings of the current study demonstrate a strong alignment between the measurement model and the collected data.

#### 4.2.2. Validity Test

This study employs convergent validity and discriminant validity to assess its validity tests [65]. Convergent validity refers to the extent of correlation observed among separate items that assess the same underlying construct [66]. The results of convergent validity are presented in Table 2. Our findings indicate that the factor loading for each measurement within the same dimension exceeds 0.5. Additionally, the combination reliability (CR) value for each variable surpasses 0.6, and the average extracted variance (AVE) value exceeds 0.5 [67]. These results suggest that the data exhibit strong convergent validity.

Discriminant validity refers to the ability to accurately differentiate and distinguish between distinct variables [65]. In this study, the software Amos 26.0 was employed to perform a discriminant validity analysis on nine factors, namely functional value (A), social value (B), emotional value (C), epistemic value (D), cultural value (E), health value (F), conditional value (G), perceived destination image (H), and revisit intention (I). The analysis aimed to validate these factors and assess the effectiveness of the measurement model (the nine-factor model) in comparison to alternative models (eight-factor, seven-factor, six-factor, five-factor, four-factor, three-factor, two-factor, and one-factor models) in terms of fitting effectiveness. The findings presented in Table 3 demonstrate that the fit indices of the nine-factor model fall within an acceptable range and are notably superior to those of the alternative models. These results suggest that the study possesses strong discriminant validity [68].

### 4.3. Structural Model and Hypotheses Testing

#### 4.3.1. Hypotheses Tests

This study employs the maximum likelihood method to assess the goodness-of-fit of the structural model. The results (χ^2^/df = 1.143, NFI = 0.935, CFI = 0.991, GFI = 0.926, AGFI = 0.913, RMSEA = 0.018, SRMR = 0.027) indicate that the goodness of fit of the structural model aligns with the statistical criteria [69].

**Figure 4:**
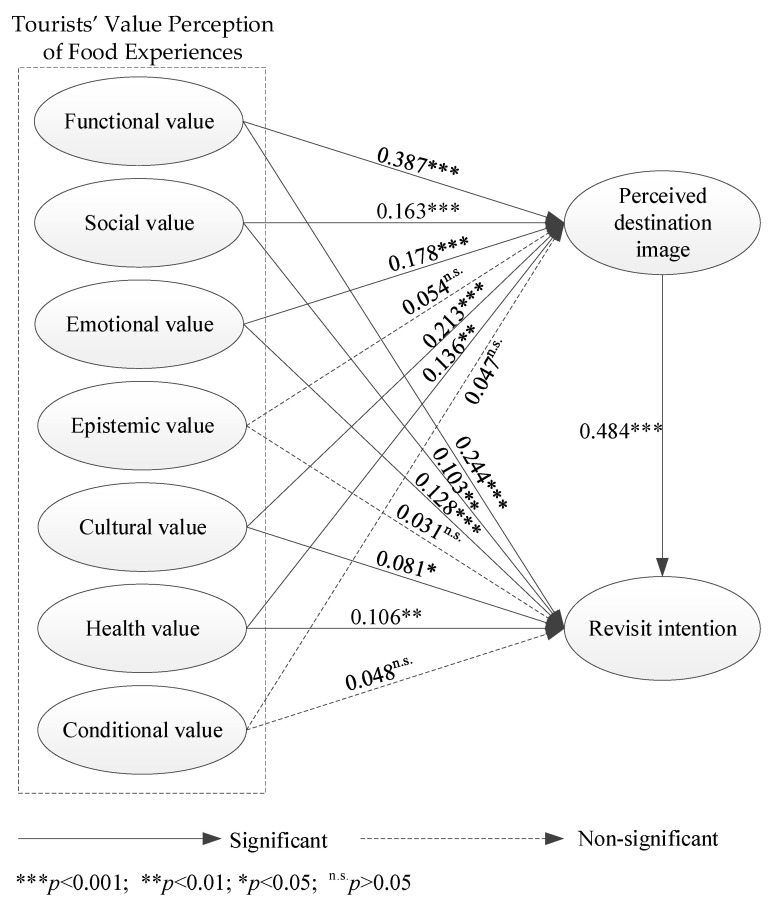
The hypothesis testing results.

The research hypotheses that were previously outlined were examined through the utilization of a structural equation model. The results of this investigation are shown in Table 4 and Figure 4. The standardized path coefficients for H1a, H1b, H1c, H1e, and H1f are 0.387, 0.163, 0.178, 0.213, and 0.316, respectively, with corresponding t-values of 7.186 (*p* < 0.001), 4.300 (*p* < 0.001), 3.953 (*p* < 0.001), 4.817 (*p* < 0.001), and 3.170 (*p* < 0.01). The results suggest that the functional, social, emotional, cultural, and health values of local food experienced by tourists have a significant positive impact on their perceived destination image. Therefore, the hypotheses H1a, H1b, H1c, H1e, and H1f were all supported.

Furthermore, the standardized path coefficients for H2a, H2b, H2c, H2e, and H2f are 0.244, 0.103, 0.128, 0.081, and 0.106, respectively. These coefficients are associated with *t*-values of 5.012 (*p* < 0.001), 3.196 (*p* < 0.01), 3.341 (*p* < 0.001), 2.173 (*p* < 0.05), and 2.966 (*p* < 0.05). The results demonstrate that the functional, social, emotional, cultural, and health values of the food experienced by tourists have a significant positive impact on their revisit intention. Therefore, the hypotheses H2a, H2b, H2c, H2e, and H2f were all supported.

In addition, the standardized path coefficients for H1d, H1g, H2d, and H2g are 0.054 (*t* = 1.375, *p* = 0.168), 0.047 (*t* = 1.301, *p* = 0.192), 0.031 (*t* = 0.969, *p* = 0.331), and 0.048 (*t* = 1.608, *p* = 0.107). This finding suggests that there is no statistically significant direct correlation between the perceived epistemic and conditional values of food, as experienced by visitors, and the perceived destination image and revisit intention. Thus, the hypotheses H1d, H1g, H2d, and H2g were not supported.

#### 4.3.2. The Mediating Role of Perceived Destination Image

In order to investigate the mediating effect of perceived destination image on the relationship between tourists’ value perceptions of food experiences and their revisit intentions, the current study utilized the bootstrap approach. The sampling process is iterated 2000 times using the selected method. The confidence interval level is set at 95%. Two specific methods, namely Bias-corrected and Percentile, are employed. The determination of the significance of the mediation effect is based on whether the output statement’s 95% confidence interval includes the value of 0 [70,71]. The results of the study are shown in Table 5. The findings indicated that the confidence intervals for the Bias-corrected and Percentile of the research hypotheses H4a, H4b, H4c, H4e, and H4f did not encompass the value of zero, and thus these hypotheses were supported. Hence, it can be observed that perceived destination image serves as a mediator in the association between tourist’s perceived functional value, social value, emotional value, cultural value, and health value of their food experience and their inclination to revisit the destination. Nevertheless, the confidence intervals for the relationships between the epistemic value, conditional value, and revisit intention include zero, suggesting that the proposed mediating effect of perceived destination image in the relationship between epistemic value, conditional value, and revisit intention is not statistically significant. Consequently, hypotheses H4d and H4g are not supported.

#### 4.3.3. The Moderating Role of Gender

The primary objective of the present study is to examine the potential moderating influence of gender within this conceptual framework. The sample (*n* = 458) was partitioned into two distinct groups: males (*n* = 232) and females (*n* = 226). A multi-group comparative analysis approach in Amos 26.0 was employed with different conditions, including the unconstrained model, measurement weights model, structural weights model, structural residuals model, and measurement residuals model. The goodness-of-fit indices for all the models that were tested are presented in Table 6.

The results of the differences between the unconstrained and constrained models are shown in Table 7. If the *p*-value is lower than 0.05, it indicates the presence of a statistically significant difference. If the *p*-value exceeds 0.05, yet the changes in the values of ΔNFI, ΔRFI, ΔIFI, ΔTLI, ΔCFI, and ΔGFI are all below 0.05, it still suggests the presence of a distinction between the models [72]. This finding indicates that gender does indeed serve as a moderator in the suggested model.

To assess the potential differences in the hypothesized paths between the male and female groups, the critical radical ratio of difference (CRD) in both samples was examined. The results are presented in Table 8. The findings suggest that there is a significant difference between the two groups in terms of the influence of the functional value (|CRD| = 3.334 > 1.96), social value (|CRD| = 2.28 > 1.96), epistemic value (|CRD| = 2.937 > 1.96), health value (|CRD| = 3.264| > 1.96), and conditional value (|CRD| = 3.168) of the food experience on perceived destination image [73]. Specifically, the effect of the functional value, social value, epistemic value, and conditional value of the food experience on perceived destination image was significantly stronger for the male group than for the female group (β = 0.459 > β = 0.163, β = 0.220 > β = 0.061, β = 0.161 > β = −0.064, β = 0.150 > β = −0.073). The impact of the health value of the food experience on the perceived destination image was lower for the male group than for the female group (β = 0.022 < β = 0.367). However, there are no moderating effects of the emotional value and cultural value of the food experience on the perceived destination image.

In addition, gender was also found to moderate the relationships of the epistemic value (|CRD| = 2.039 > 1.96) and cultural value (|CRD| = 2.84 > 1.96) of the food experience to revisit intention. Epistemic value indicated a greater impact on revisit intention for the male group (β = 0.108) than the female group (β = −0.229). However, the impact of the cultural value on revisit intention for the male group (β = −0.005) was lower than for the female group (β = −0.233). There are no moderating effects of the functional value, social value, emotional value, health value, and conditional value of the food experience on revisit intention. Therefore, H5a, H5b, H5d, H5f, H5g, H6d, and H6e were supported, while H5c, H5e, H6a, H6b, H6c, H6f, and H6g were not supported.

## 5. Discussion

### 5.1. Theoretical Implications

The present study offers multiple noteworthy contributions to the existing body of literature. First, it represents one of the first attempts to empirically examine the interrelationships between tourists’ value perceptions of food experiences, perceived destination images, and their revisit intention. Particularly, this study investigated the impacts of the multi-dimensional value perceptions of food experiences on tourists’ perceived destination images and revisit intentions. Based on the theory of consumption value, this study measured tourists’ value perceptions of food experiences through seven distinct dimensions, namely functional value, social value, emotional value, epistemic value, cultural value, health value, and conditional value [10,15,17]. The structural model exhibits a high level of congruence and demonstrates robust plausibility. The findings are aligned with the study of Hsu et al. [19]. The results indicate that the value perceptions of dimensions of food, such as health and emotional value, contribute to the perceived destination image. This discovery is also supported by the studies of Kim et al. [74] and Gupta et al. [75], and provides a strong foundation for future investigations that utilize these specific dimensions of food.

Second, this study investigated the mediating effects of perceived destination image and examined tourists’ perceptions of a destination’s image during their journeys. The majority of existing studies have concentrated on the perceptions of potential tourists regarding the image of a destination prior to their actual journey [18,76]. Nevertheless, given they have not physically reached the destination, their impression of the destination’s image may be influenced by subjective biases. This finding is in line with the research of Yeap et al. [77] and Shin et al. [78] and provides an illustration of the conclusion of the differential relationship between tourists’ value perceptions of food experiences and their willingness to revisit that has been seen in previous food tourism research. The current study investigated the way tourists perceive the image of a destination while they are actually experiencing tourism activities. The study also explored the role of destination image as a mediating factor. The findings revealed that the functional, social, emotional, cultural, and health values of local food experienced by tourists have significant positive impacts on their perceived destination image, and perceived destination image partially mediated the influence of tourists’ value perceptions of food experiences on their revisit intention, which is consistent with the findings of several other studies in this field [19,79]. Furthermore, this study unequivocally validates that tourists’ perceived destination images can have a significant positive effect on their intention to revisit, and the more positive the perceived destination image is, the more likely they are to revisit and recommend the destination to their friends and family [18,19,80].

Third, the present study employed a multi-group analysis method to analyze the moderating effects of tourists’ genders, which expands the scope of research on tourists’ value perceptions of food experiences and their behavior, and addresses our inquiry based on the existing body of research [81]. The findings indicate that gender plays a moderating role in the direct effects of the tourists’ perceived epistemic and cultural values of their food experiences on their intentions to revisit a destination. Additionally, gender also moderates the impact of functional, social, cognitive, health, and condition values of the food experience on the perceived destination image. Prior research has identified disparities in food consumption patterns based on gender, as men and women exhibit distinct dietary needs and considerations [82]. This study offers a more specific example of this perspective, particularly regarding the greater impact of men’s perceived functional, social, epistemic, and conditional values of food experiences on their destination image compared to women’s. Additionally, men perceived value have a stronger influence on their intentions to revisit. Regarding the impact of tourists’ perceptions of the health value of food on their perceived destination image, as well as the influence of the cultural value of food on their intention to revisit, women exhibit a greater strength in these areas than men. The belief that women are more capable of exercising weight control and possessing nutritional knowledge than men could account for the fact that women hold more firm convictions regarding healthful eating [83,84,85].

### 5.2. Practical Implications

This study provides valuable practical implications for tourism destination managers and marketers. First, more emphasis must be placed on improving the overall quality of local food, which is essential in order to provide tourists with delectable tastes and enticing flavors. The findings of this study demonstrate that the multi-dimensional value perceptions of food experiences have great impacts on tourists’ perceived destination images and revisit intentions. Thus, providing high-quality local food requires paying great attention to tourists’ value perceptions of their food experiences. In order to offer tourists exceptional local food, destination managers and marketers must effectively convey the various aspects of the food’s value to food producers, such as hotels, restaurants, and chefs. In addition, destination managers and marketers should promote the incorporation of local gastronomy culture into tourists’ travel experiences in order to enhance the appeal of local food and spread the local food culture.

Second, destination managers should strengthen the support for well-established food brands that can represent the image of the destination, and take the initiative to uncover food stories and legends with historical and cultural value, and combine them with the publicity and promotion of food tourism. Our empirical results indicate that the cultural values of local food experienced by tourists have a significant positive impact on their perceived destination image and revisit intention. Hence, conducting thorough research on traditional food brands that can effectively embody a destination’s identity can have a dual impact. Firstly, they can enhance the destination’s brand image. Secondly, they can stimulate tourists’ inclination to recommend and revisit, thereby fostering the sustainable development of the destination’s socio-economic sector. It is advisable for practitioners in these destinations to offer food tourists an opportunity to observe the food preparation process and incorporate it into their perception of the local culture [86]. For instance, implementing regular food-themed experiential activities to actively engage travelers and enhance the quality of their tourism experience. This will create an engaging and interactive experience for tourists.

Third, local food marketing for destinations should have different focuses for various gender demographics of tourists. The current study found that the impact of functional value, social value, epistemic value, and conditional value of food experiences on the perceived destination image was notably greater in the male group compared to the female group. However, the influence of health value on the perceived image of the location was less significant in the male group compared to the female group. Additionally, epistemic value had a greater impact on revisit intention for the male group than the female group, while the impact of cultural value on revisit intention for the male group was lower than for the female group. Hence, when promoting local food in a particular destination, it is important to highlight the health and cultural significance of the food experience for female tourists, as well as the functional value, social value, epistemic value, and conditional value of the food experience for male tourists.

## 6. Conclusions

This study empirically examines the impact of tourists’ value perceptions of food experiences on their perceived destination image and revisit intention, and explores the moderating effects of gender on the relationships between tourists’ value perceptions of food experiences, perceived destination image, and their revisit intentions. Nanjing Impressions was chosen as the research case. A total of 500 questionnaires were distributed and collected. A sample size of 458 was eligible for analysis. The findings show that the functional, social, emotional, cultural, and health values of local food experienced by tourists have significant positive impacts on their perceived destination image and revisit intention. The perceived destination image of tourists has positive and significant influence on their revisit intention. Moreover, perceived destination image serves as a mediator in the association between tourist’s perceived functional value, social value, emotional value, cultural value, and health value of food they experienced and their inclination to revisit the destination. In addition, gender was found to play a role as a moderating variable in the previously described relationship. Male tourists place greater emphasis on the functional value, social value, epistemic value, and conditional value of food tourism experiences, whereas female tourists prioritize the health value and cultural value. This study offers valuable practical insights for destination managers. On the one hand, it is important to prioritize the quality of local food, enhance the promotion of food brands that embody the local identity, and delve into the narratives and historical significance of local food. On the other hand, distinct promotional approaches ought to be implemented for different genders of tourists. For instance, emphasis should be placed on the cultural and health benefits associated with the food experience for female tourist groups.

The current work has established a strong foundation for future scholarly investigations. However, it is essential to acknowledge the inherent limitations of this study. First, this study solely relied on a single research case, Nanjing Impressions. Subsequent research could incorporate a wider range of traditional food brands that can represent the destination image under empirical examination, thereby verifying the findings of this study. Second, this study exclusively investigated the cultural setting inside China, and while there may be some variation among provinces, the general depiction of culture is largely consistent. It is suggested that future research could expand this study by examining different cultural backgrounds, such as comparing Western and Chinese cultures. Finally, relationships between tourists’ value perceptions of food experiences, their perceived destination image, and their revisit intention may vary based on the social demographics of tourists. The present study only investigated the moderating effects of gender on the aforementioned relationships. Future research could explore the moderating role of other sociodemographic variables such as age, income, and occupation.

## Figures and Tables

**Table 1:** The demographic characteristics of the sample.

		Frequency (*n* = 458)	Percentage (%)
Gender	Male	232	50.7
	Female	226	49.3
Age	18 to 22 years	79	17.2
	23 to 35 years	144	31.4
	36 to 45 years	105	22.9
	46 to 55 years	75	16.4
	56 to 65 years	41	9.0
	Over 65 years	14	3.1
Education	High school or below	60	13.1
	Associate degree	148	32.3
	Bachelor’s degree	206	45.0
	Master’s degree or above	44	9.6
Occupation	Company employee	201	43.9
	Self-employment or owner	60	13.1
	Student	76	16.6
	Government official	49	10.7
	Professional, teacher, or technical	39	8.5
	Personnel in farming, forestry, livestock, or fishing	19	4.1
	Other	14	3.1
Personal monthly income (CNY)	Less than 3000	87	19.0
	3001–4500	13	2.8
	4501–6000	74	16.2
	6001–7500	165	36.0
	7501–10,000	82	17.9
	10,001–15,000	23	5.0
	More than 15,000	14	3.1

**Table 2:** Reliability and confirmatory factor analysis results.

Variables	Mean (SD)	Factor Loading	CR	AVE	Cronbach’s α
**Functional value (FV)**			0.832	0.554	0.831
It is convenient	3.50 (1.15)	0.74			
The service is quick.	3.48 (1.10)	0.73			
There is a variety of menu choice.	3.48 (1.06)	0.72			
The food portion is enough to satisfy my hunger.	3.53 (1.04)	0.78			
**Social value (SV)**			0.865	0.681	0.864
Having the food in Nanjing Impressions obtain social approval.	3.36 (1.15)	0.78			
Many people that I know used to go to the restaurant.	3.29 (1.20)	0.86			
The customers in the restaurant or having the food have certain level and style.	3.32 (1.11)	0.84			
**Emotional value (EmV)**			0.880	0.646	0.879
The foods in the restaurant can make me feel happy	3.56 (1.21)	0.83			
The foods in the restaurant can give me pleasure.	3.49 (1.15)	0.77			
The foods in the restaurant make me feel excited.	3.45 (1.11)	0.79			
The foods in the restaurant make me crave it.	3.53 (1.20)	0.83			
**Epistemic value (EpV)**			0.853	0.660	0.852
I want to seek out more information about foods in Nanjing.	3.64 (1.05)	0.77			
I want to try more diverse foods in Nanjing	3.64 (1.06)	0.86			
It is a good opportunity for me to learn something new about the food in Nanjing.	3.61 (1.06)	0.80			
**Cultural value (CuV)**			0.907	0.583	0.907
It enables me to learn what this local food tastes like.	3.58 (1.14)	0.76			
It provides authentic experience.	3.50 (1.13)	0.73			
It helps me see how local people live.	3.43 (1.04)	0.76			
It makes me see the things that I don’t normally see.	3.50 (1.06)	0.73			
It offers a unique opportunity to understand local cultures.	3.45 (1.08)	0.71			
It provides special dining experience in its traditional setting.	3.46 (1.06)	0.80			
It gives me an opportunity to increase my knowledge about different cultures.	3.47 (1.12)	0.86			
**Health value (HV)**			0.885	0.659	0.885
The foods in the restaurant are hygienic.	3.59 (1.02)	0.83			
The foods in the restaurant are safe.	3.72 (1.11)	0.81			
The foods and restaurant make me healthy.	3.62 (1.09)	0.80			
The foods in the restaurant can provide me with good nutrition.	3.66 (1.07)	0.80			
**Conditional value (CoV)**			0.890	0.802	0.886
I would try the Nanjing salted duck	3.49 (1.07)	0.95			
I would try the vermicelli cooked with duck blood	3.53 (1.09)	0.84			
**Perceived destination image (PDI)**			0.925	0.607	0.925
Nanjing is a city full of opportunity for adventure	3.64 (1.04)	0.78			
Nanjing is rich in exciting nightlife and entertainment	3.68 (1.02)	0.77			
Nanjing is relaxing	3.66 (1.07)	0.78			
Nanjing is pleasant	3.78 (1.05)	0.80			
The residents of Nanjing are friendly and trustworthy	3.69 (1.03)	0.78			
The residents of Nanjing are communicative	3.72 (1.03)	0.79			
Nanjing is very safe	3.67 (1.01)	0.78			
Nanjing has attractive tourist sightseeings and activities	3.67 (0.97)	0.74			
**Revisit intention (RI)**			0.858	0.669	0.857
I’d love to come to Nanjing again	3.81 (1.02)	0.86			
I will come back to Nanjing in near future	3.77 (1.00)	0.79			
I will encourage family and friends to visit Nanjing	3.71 (1.00)	0.80			

Note: Model fit indices: χ^2^/df = 1.141, NFI = 0.935, CFI = 0.991, TLI = 0.990, GFI = 0.926, AGFI = 0.913, RMSEA = 0.018, SRMR = 0.027. All the factor loads are greater than 0.5, and the *p* values are significant (*p* < 0.001).

**Table 3:** The model fit results.

Model	χ^2^/df (<3)	GFI (>0.9)	RMSEA (<0.10)	RMR (<0.05)	CFI (>0.9)	NFI (>0.9)	NNFI (>0.9)
Nine factors (A, B, C, D, E, F, G, H, I)	1.143	0.926	0.018	0.031	0.991	0.935	0.990
Eight factors (A + B, C, D, E, F, G, H, I)	2.073	0.865	0.048	0.058	0.934	0.880	0.927
Seven factors (A + B + C, D, E, F, G, H, I)	3.075	0.780	0.067	0.078	0.870	0.820	0.859
Six factors (A + B + C + D, E, F, G, H, I)	3.884	0.744	0.079	0.089	0.818	0.771	0.803
Five factors (A + B + C + D + E, F, G, H, I)	5.065	0.660	0.094	0.115	0.742	0.699	0.723
Four factors (A + B + C + D + E + F, G, H, I)	6.149	0.609	0.106	0.119	0.671	0.632	0.649
Three factors (A + B + C + D + E + F + G, H, I)	6.792	0.595	0.113	0.123	0.628	0.592	0.605
Two factors (A + B + C + D + E + F + G + H, I)	7.509	0.568	0.119	0.125	0.581	0.547	0.556
One factor (A + B + C + D + E + F + G + H + I)	7.567	0.567	0.120	0.125	0.577	0.543	0.552

**Table 4:** Standardization path coefficients and hypothesis testing results.

Hypothesis Paths	Estimate	S.E.	*t*	*p*	Results
H1a: FV ⟶ PDI	0.387	0.048	7.186	***	Supported
H1b: SV ⟶ PDI	0.163	0.032	4.300	***	Supported
H1c: EmV ⟶ PDI	0.178	0.037	3.953	***	Supported
H1d: EpV ⟶ PDI	0.054	0.038	1.375	0.168	Not supported
H1e: CuV ⟶ PDI	0.213	0.037	4.817	***	Supported
H1f: HV ⟶ PDI	0.136	0.042	3.170	0.001 **	Supported
H1g: CoV ⟶ PDI	0.047	0.033	1.301	0.192	Not supported
H2a: FV ⟶ RI	0.244	0.047	5.012	***	Supported
H2b: SV ⟶ RI	0.103	0.029	3.196	0.001 **	Supported
H2c: EmV ⟶ RI	0.128	0.033	3.341	***	Supported
H2d: EpV ⟶ RI	0.031	0.034	0.969	0.331	Not supported
H2e: CuV ⟶ RI	0.081	0.033	2.173	0.029 *	Supported
H2f: HV ⟶ RI	0.106	0.038	2.966	0.003 **	Supported
H2g: CoV ⟶ RI	0.048	0.029	1.608	0.107	Not supported
H3: DI ⟶ RI	0.484	0.061	8.542	***	Supported

Note: *** *p* < 0.001, ** *p* < 0.01, * *p* < 0.05.

**Table 5:** Results of mediation effect analysis.

Hypothesis Paths	Point Estimate	Product of Coefficients		Bias-Corrected 95% CI		Percentile 95% CI	
		SE	Z (≥1.96)	Lower	Upper	Lower	Upper
H4a: FV ⟶ PDI ⟶ RI	0.214	0.043	4.977	0.119	0.263	0.115	0.257
H4b: SV ⟶ PDI ⟶ RI	0.080	0.023	3.478	0.037	0.121	0.034	0.113
H4c: EmV ⟶ PDI ⟶ RI	0.076	0.022	3.455	0.037	0.128	0.034	0.123
H4d: EpV ⟶ PDI ⟶ RI	0.028	0.022	1.273	−0.012	0.075	−0.015	0.072
H4e: CuV ⟶ PDI ⟶ RI	0.106	0.027	3.926	0.051	0.148	0.047	0.142
H4f: HV ⟶ PDI ⟶ RI	0.065	0.026	2.500	0.023	0.137	0.017	0.127
H4g: CoV ⟶ PDI ⟶ RI	0.019	0.016	1.188	−0.011	0.057	−0.014	0.055

**Table 6:** Goodness of fit indices for tested models.

Model	χ^2^	df	χ^2^/df (<2.00)	*p*	TLI (>0.90)	CFI (>0.90)	IFI (>0.90)	RMSEA (<0.05)	AIC	ECVI
Unconstrained	1515.574	1258	1.205	0.000	0.972	0.975	0.976	0.021	1963.574	4.306
Measurement weights	1552.156	1287	1.206	0.000	0.972	0.974	0.975	0.021	1942.156	4.259
Structural weights	1648.182	1302	1.266	0.000	0.964	0.967	0.967	0.024	2008.182	4.404
Structural residuals	1884.074	1332	1.414	0.000	0.944	0.947	0.947	0.030	2185.374	4.792
Measurement residuals	1972.204	1370	1.44	0.000	0.94	0.942	0.942	0.031	2184.074	4.79

**Table 7:** Significance of tested models compared to the unconstrained model.

Model	Δχ^2^	Δdf	*p*	ΔNFI	ΔRFI	ΔIFI	ΔTLI	ΔCFI	ΔGFI	ΔAGFI
Measurement weights	36.582	29	0.160	−0.003	0.000	−0.001	0.000	−0.001	−0.003	0.000
Structural weights	132.608	44	0.000	−0.011	−0.007	−0.009	−0.008	−0.008	−0.010	−0.006
Structural residuals	368.500	74	0.000	−0.031	−0.025	−0.029	−0.028	−0.028	−0.026	−0.020
Measurement residuals	456.630	112	0.000	−0.039	−0.028	−0.034	−0.032	−0.033	−0.032	−0.021

**Table 8:** Path coefficient comparisons between male and female groups.

Hypothesis Paths	Male Group			Female Group			|CRD|
	Estimate	*t*	*p*	Estimate	*t*	*p*	
H5a: FV ⟶ PDI	0.459	6.036	***	0.163	2.448	0.014 *	3.334
H5b: SV ⟶ PDI	0.220	3.422	***	0.061	1.299	0.194	2.280
H5c: EmV ⟶ PDI	0.093	1.812	0.070	0.185	2.241	0.025 *	0.822
H5d: EpV ⟶ PDI	0.161	2.658	0.008 **	−0.064	−1.358	0.174	2.937
H5e: CuV ⟶ PDI	0.090	1.785	0.074	0.263	3.102	0.002 **	1.613
H5f: HV ⟶ PDI	0.022	0.427	0.669	0.367	3.705	***	3.264
H5g: CoV ⟶ PDI	0.150	2.818	0.005 **	−0.073	−1.57	0.116	3.168
H6a: FV ⟶ RI	0.216	2.766	0.006 **	0.244	4.245	***	0.310
H6b: SV ⟶ RI	0.169	2.859	0.004 **	0.045	1.157	0.247	1.843
H6c: EmV ⟶ RI	0.075	1.641	0.101	0.151	2.202	0.028 *	0.944
H6d: EpV ⟶ RI	0.108	1.99	0.047 *	−0.029	−0.75	0.453	2.039
H6e: CuV ⟶ RI	−0.005	−0.111	0.912	0.233	3.265	0.001 **	2.840
H6f: HV ⟶ RI	0.050	1.111	0.267	0.21	2.387	0.017 *	1.869
H6g: CoV ⟶ RI	0.101	2.079	0.038 *	0.019	0.486	0.627	1.377

Note: *** *p* < 0.001, ** *p* < 0.01, * *p* < 0.05.

## Data Availability

The data presented in this study are available on request from the corresponding author. Data is contained within the article.
